# CXCL5 drives obesity to diabetes, and further

**DOI:** 10.18632/aging.100064

**Published:** 2009-07-02

**Authors:** Carine Chavey, Lluis Fajas

**Affiliations:** IRCM, Institut de Recherche en Cancérologie de Montpellier, Montpellier, F-34298, France; INSERM, U896, Montpellier, F-34298, France; Université de Montpellier1, Montpellier, F-34298, France

**Keywords:** chemokine, adipose tissue, obesity, diabetes

## Abstract

We have recently shown that the CXCL5 chemokine is secreted by adipose tissue in the obese state. We demonstrated that adipose tissue-derived CXCL5 mediates insulin resistance in muscle. We speculate in this paper that CXCL5 could also mediate other obesity, and diabetes-derived pathologies, such as cardiovascular disease, retinopathy, or inflammatory bowel disease. In this scenario CXCL5 targeted therapy would prevent not only the development of type II diabetes in obese subjects, but also several other obesity-related co morbidities. Finally we propose to analyze the CXCL5 gene to find particular polymorphisms that could predict the development of type II diabetes in obese subjects.

The major environmental risk factors for type II diabetes  are obesity and a sedentary lifestyle [1], and the dramatic increase in the rates of type II diabetes in recent years has been attributed, primarily, to the striking rise in obesity worldwide [2]. Adipose tissue is absolutely required for glucose homeostasis. Indeed, subjects with lipoatrophy and transgenic animals that are engineered to lack adipose tissue are extremely insulin resistant [3]. This seems therefore to indicate that storage of energy in adipocytes favors insulin sensitivity. Adipose tissue dysfunction, which is associated with obesity is the key factor of obesity-related insulin resistance and type II diabetes. Since adipose tissue only contributes minimally to glucose disposal, signaling pathways might exist from adipose tissue to muscle and other insulin sensitive tissues. Both proteins and lipids have been proposed as non-mutually exclusive signaling molecules, which can affect the muscle. A first group of important mediators consists of fatty acids. Since the original observation by Randle, it has been established that increased fatty acid concentrations in the muscle decrease glucose metabolism (reviewed in [4]).  A second class of mediators that affect insulin sensitivity in both muscle and liver and which are derived from adipose tissue are adipokines. Adipokines are factors secreted by the different cell compartments of white adipose tissue (WAT), such as adipocytes or macrophages, and were initially characterized as regulators of metabolic processes, such as regulation of food intake, energy homeostasis, adipocyte differentiation, or insulin sensitivity.  Subsequently, it was found that adipokines could modulate inflammatory processes. These adipokines include WAT-specific factors, such as leptin, adiponectin, and well-known cytokines secreted by several cell types, such as TNF-alpha, IL-6, IL-8, IL-1, or monocyte chemoattractant protein-1 [5]. In our recent publication we identify the CXCL5 chemokine as one of these signaling molecules secreted in adipose tissue that have major implications in insulin sensitivity in muscle cells [6].

CXCL5 or epithelial neutrophil activating peptide (ENA-78) is a cytokine belonging to the family of chemokines that is mainly implicated in the chemotaxis of inflammatory cells through the generation of local concentration gradients [7,8]. It has been shown to be a recruiter of neutrophils and involved in their activation. This C-X-C chemokine has been implicated in pulmonary disease, lung cancer, arthritis, and other pathological states [7,9,10]. In our paper we show that CXCL5 is a new chemokine secreted by adipose tissue resident macrophages and that circulating CXCL5 is highly increased during obesity in both mice and humans. CXCL5 is able to inhibit insulin action in muscle by activating the Jak/STAT/SOC signaling pathway showing that CXCL5 can induce insulin resistance. Higher CXCL5 level is associated with insulin-resistant patients compared to non-insulin-resistant obese patients. Moreover, CXCL5 is directly regulated by TNFα in both adipose tissue and macrophages by NFκB activation, suggesting that CXCL5 mediates the effects of TNFα in insulin resistance. Most importantly, inhibition of signaling from CXCR2, which is the CXCL5 receptor, by injection of neutralizing anti-CXCL5 antibody or selective antagonist to CXCR2 in insulin-resistant-obese mice improves both insulin sensitivity and glucose clearance. In summary our data show that CXCL5 promotes insulin resistance [6].

**Figure 1.**
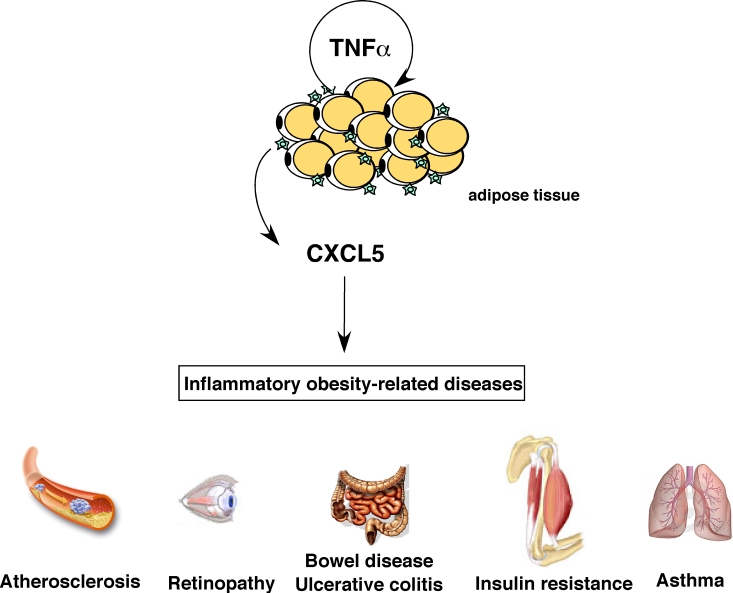
Role of CXCL5 in inflammatory obesity-related pathologies. CXCL5 is produced in response to TNFα by adipose tissue-resident macrophages and can trigger several obesity-associated complications like asthma, atherosclerosis, bowel disease, colitis, diabetes and retinopathy.

## Implication of CXCL5 in other pathological conditions associated to obesity-induced diabetes

In addition to insulin resistance, obese diabetic patients are at high risk to develop associated pathologies, including, but not limited to atherosclerosis, retinopathies, or other inflammatory diseases. This is represented in figure 1. Interestingly, a major common feature of these pathologies is inflammation. Since CXCL5 is an inflammatory factor, and since its levels are increased in obese patients, we could speculate that CXCL5 is at the origin of obesity- associated co-morbidities. Furthermore, the CXCL5 receptor CXCR2 is expressed in cells other than muscle cells, such as endothelial, pulmonary, or intestinal epithelial cells. In this context, it is interesting the recently suggested correlation between obesity and asthma [11]. Strikingly, exacerbation of asthma has been also correlated with increased expression of both CXCL5 and its receptor CXCR2 [12].

Atherosclerosis is another obesity-related risk factor in which CXCR2 could play an important role. This receptor is found in macrophage-rich intimae in human atherosclerotic lesions, and it has been shown to have a major impact on macrophage accumulation in advanced lesions [13]. CXCR2 ligands, such as GRO-α participate in this macrophage accumulation and lesion progression, although they might not have a causative role [14], but rather contribute to disease progression. CXCL5 could also participate in this process.

Secondary to obesity-induced diabetes is the development of retinopathy. Development of diabetic retinopathy is a multifactorial process, and affects as much as 30% of type II diabetic patients. Much of the damage of retinopathy results from leakage of retinal blood vessels and inadequate retinal perfusion. [15] Sustained hyperglycemia in diabetes affects various vasoactive factors, such as vascular endothelial growth factor [16]. These factors, which are all interrelated, contribute to development of structural and functional changes in diabetic retinopathy, such as breakdown of the blood-retina barrier. Participation of CXCL5 in the development of retinopathy was suggested by the increased levels of this chemokine found in retinopathy diabetic patients [17].

Finally, but not limited to, CXCL5 could be also involved in the development of obesity-related inflammatory bowel disease. Although obesity has not been directly linked to the pathophysiology of inflammatory bowel disease (IBD), increased macrophage numbers as well as enhanced production of proinflammatory adipokines in obese patients may create a favorable environment for disease progression in intestinal inflammation and IBD [18]. Increased basal cytokine levels associated with obesity, both due to increased adipocytes numbers and size may predispose to more severe outcomes in IBD patients. Recent observations indicating that fat tissue is also associated with immune responses also suggest a link between obesity and gut inflammation [19].  The proinflammatory effects of CXCL5 are widely accepted. Furthermore, it was shown that CXCR2 plays a crucial pathophysiological role in experimental ulcerative colitis in mice [20]. In humans, a marked increase in ENA-78 has been reported in ulcerative colitis patients [21], and has been shown to be localized to colonic epithelial cells in IBD tissues [21,22]. Taken together, these observations suggest that the increased CXCL5 circulating levels observed during obesity could contribute to the development or progression of IBD.

Studies aiming to elucidate the role of WAT-secreted CXCL5 in all these obesity-related pathologies are likely to be forthcoming in the near future. Inhibiting CXCL5 secretion or function in obese individuals not only ameliorate their insulin sensitivity, but could also decrease the risk of developing other major obesity-related pathologies.

## CXCL5 gene polymorphisms

It is now accepted that type II diabetes is, in part, inherited.  Family studies have revealed that first degree relatives of individuals with type II diabetes are about 3 times more likely to develop the disease than individuals without a positive family history of the disease [23].  It has also been shown that concordance rates for monozygotic twins, which have ranged from 60-90%, are significantly higher than those for dizygotic twins.  It is therefore clear that type II diabetes has a strong genetic component. Candidate genes identified sofar include the nuclear receptor PPARγ, the sulfonylurea receptor ABCC8, the potassium channel Kir6.2, or the intracellular calcium-dependent cystein protease calpain 10. Taking into account the relative importance of CXCL5 in the development of insulin resistance we can hypothesize that this chemokine could also be a type II diabetes susceptibility gene. Indeed several polymorphisms in the CXCL5 gene have been described. Interestingly, a -156G to C polymorphism in the promoter of the gene has been associated to increased expression and plasma concentration of CXCL5. It cannot be excluded that this or other activating polymorphisms are overrepresented in type II diabetes and obese subjects.

## Anti CXCL5-CXCR2 based therapies

Despite the list of new and classical agents designed for the treatment of type II diabetes, such as thiazolidinediones, biguanides, meglitinides, or sulphonylureas is increasingly long, a major challenge remains because even using the more aggressive therapy, glycemic control in type II diabetic patients may still deteriorate. Our study may provide a new therapeutic target. We show that inhibition of the CXCL5-CXCR2 axis, both by CXCR2 antagonists or CXCL5 blocking antibodies decreases glycemia in mice models of diabetes. Long term treatments are currently being evaluated in our laboratory. The most interesting feature of this newly identified target is that is directed not only for insulin resistance treatment, but could also target diabetes-associated co-morbidities. It is interesting to notice, at some extent, similarities between other insulin-sensitizing drugs, such as metformin and anti-CXCL5 therapy. Similar to metformin, CXCL5 antagonism restores insulin sensitivity, has anti-atherosclerosis effects, and could be even beneficial as anti-cancer agent.  From this perspective, anti-CXCL5 therapy could be also considered as anti-aging therapy (reviewed in [24]). Safety studies of the tested molecules, as well as discovery of new CXCR2 antagonists are guaranteed.
